# Optimized BEAC conditioning regimen improves clinical outcomes of autologous hematopoietic stem cell transplantation in non-Hodgkin lymphomas

**DOI:** 10.1007/s12185-024-03755-7

**Published:** 2024-04-08

**Authors:** Sha Zhou, Jun Rao, Xiangyu Ma, Yunjing Zeng, Xixi Xiang, Jiali Li, Hongyun Liu, Shijia Lin, Song Dong, Fu Li, Xi Zhang, Li Gao

**Affiliations:** 1 Medical Center of Hematology, Xinqiao Hospital of Army Medical University, State Key Laboratory of Trauma and Chemical Poisoning, Chongqing Key Laboratory of Hematology and Microenvironment, Chongqing, China; 2 National Clinical Research Center for Hematologic Diseases, the First Affiliated Hospital of Soochow University, Suzhou, Jiangsu China; 3 Department of Epidemiology, Army Medical University, Chongqing, China

**Keywords:** Non-Hodgkin lymphoma, Conditioning regimen, Autologous hematopoietic stem cell transplantation, BEAC

## Abstract

The conditioning regimen is an important part of autologous hematopoietic stem cell transplantation (ASCT). We explored the efficacy and safety of an optimized BEAC (adjusted-dose, intermediate-dose cytarabine and reduced-dose cyclophosphamide, AD-BEAC) conditioning regimen for non-Hodgkin lymphoma (NHL). A total of 141 NHL patients received AD-BEAC or a standard-dose BEAC (SD-BEAC) conditioning regimen from January 2007 to December 2017, and 104 patients were included in the study after 1:1 propensity matching. The 5-year overall survival (OS) and progression free survival (PFS) rates were significantly higher with AD-BEAC than with SD-BEAC (82.7% vs. 67.3%, *P* = 0.039; 76.9% vs. 57.7%, *P* = 0.039). Transplant-related mortality (TRM) was 3.8% in both the AD-BEAC and SD-BEAC groups. The AD-BEAC group had lower incidence of oral ulcers and cardiotoxicity than the SD-BEAC group. An optimized BEAC conditioning regimen is an effective conditioning regimen for ASCT in NHL with acceptable toxicity, that is more effective and safer than a standard BEAC conditioning regimen.

**Supplementary Information:**

The online version contains supplementary material available at 10.1007/s12185-024-03755-7.

## Introduction

High-dose chemotherapy (HDT) followed by autologous hematopoietic stem cell transplantation (ASCT) is a standard therapy for patients with relapse/refractory lymphoma, and high-risk non-Hodgkin lymphoma (NHL) patients also benefit from first-line ASCT [1]. Conditioning regimens play an important role in ASCT. Over the last few decades, many different conditioning regimens have been used in lymphoma transplant; these regimens can be divided into two types: total body irradiation (TBI)-based regimens and chemotherapy-only regimens. TBI-based conditioning regimens are associated with a higher incidence of second malignancies, which is accompanied by increased non-relapsed mortality (NRM) [2]. Therefore, many transplant centers use chemotherapy-only regimens without TBI, such as BEAM, BEAC, CBV and BuCy, and BEAM and BEAC regimens are most widely used [3, 4].

It is important to find a conditioning regimen for NHL that is effective with low toxicity. High-dose cytarabine-containing (total dose 2000–4000 mg/m^2^) therapy, including DHAP and ESHAOx regimens, has been shown to be an effective regimen that is non-cross-resistant in patients with relapsed or refractory lymphoma [5, 6]. On the other hand, in the dose study of CY [7], the dose of CY administered ranged from 750 to 2350 mg/m^2^/d. Cardiotoxicity was significantly more likely to occur at a CY dose of > 1550 g/m^2^/d, but a lower dose did not compromise the transplantation effect. CY 1500 mg/m^2^/d as a part of the BEAC conditioning regimen can lead to very acute cardiac toxicity in NHL patients previously treated with anthracyclines [8]. Based on these findings, we thought that increasing the dose of cytarabine and reducing the dose of cyclophosphamide in the BEAC conditioning regimen might further improve its efficacy and reduce its toxicity.

In the BEAC regimen, cardiotoxicity is potentially the most threatening nonhematological side effect of high-dose cyclophosphamide (CY) [5]. Previous studies have shown that high-dose CY results in very acute cardiac toxicity characterized by enlargement of the heart chambers in NHL patients previously treated with anthracyclines [5, 6]. High-dose cytarabine-containing therapy, including DHAP and ESHAOx regimens, has been shown to be an effective regimen that is non-cross-resistant in patients with relapsed or refractory lymphoma [7, 8]. Therefore, reducing the dose of cyclophosphamide and increasing the dose of cytarabine in the BEAC conditioning regimen may further improve its efficacy and reduce its toxicity. We explored an optimized BEAC (adjusted-dose BEAC, AD-BEAC) conditioning regimen with the dose of cyclophosphamide reduced from 1.5 g/m^2^ to 1 g/m^2^ and the dose of cytarabine increased from 200 to 1000 mg/m^2^. In the present study, we retrospectively analyzed NHL patients who received ASCT with an AD-BEAC or standard-dose BEAC (SD-BEAC) conditioning regimen at Xinqiao Hospital in the past 11 years to confirm whether adjusting the dose of the BEAC conditioning regimen is beneficial.

## Materials and methods

### Study design and patient selection

We retrospectively analyzed patients with NHL who underwent ASCT with a BEAC conditioning regimen from January 2007 to December 2017 at Xinqiao Hospital; 82 patients received an AD-BEAC regimen, and 59 patients received a SD-BEAC regimen. The inclusion criteria were as follows: NHL patients aged 18–70 years with adequate cardiac, hepatic, and renal function prior to transplantation; eastern cooperative oncology group (ECOG) performance status of 0-2; and newly diagnosed high-risk NHL or relapsed/refractory NHL achieving complete remission (CR) or partial remission (PR). Our study was approved by the Institutional Review Board and was conducted in accordance with the declaration of Helsinki. The patients provided written informed consent prior to participating in the study. To avoid a possible imbalance, propensity score matching was applied, and the propensity score was obtained from a logistic regression model. The covariates were age, gender, lymphoma type, stage, time from diagnosis to transplant, chemotherapy cycles before transplant, disease status at transplant, and newly diagnosed or relapsed/refractory NHL. The AD-BEAC group cases were analyzed by intention-to-treat and matched in a 1:1 ratio to SD-BEAC controls based on the propensity score with a standard caliper width of 0.2. A total of 104 patients were included in the study.

### Conditioning regimen and supportive care

The AD-BEAC regimen comprised 300 mg/m^2^ × 1 day [d] of carmustine, 200 mg/m^2^ × 4 d of etoposide, 1000 mg/m^2^ × 4 d of cytarabine, and 1 g/m^2^ × 4 d of cyclophosphamide. The SD-BEAC regimen included 300 mg/m^2^ × 1 d of carmustine, 200 mg/m^2^ × 4 d of etoposide, 200 mg/m^2^ × 4 d of cytarabine, and 1.5 g/m^2^ × 4 d of cyclophosphamide [9]. Stem cells were infused 48 h after the last dose of the regimen. All patients received prophylaxes for bacterial infection, fungal infection, herpes simplex virus infection, and pneumocystis pneumonia, as in our previous work [10, 11].

### Definition and outcome evaluation

The response criteria were based on the 2007 Revised Response Criteria for Malignant Lymphoma [12]. The toxicity assessment was performed according to the common terminology criteria for adverse events (CTCAE), version 4.0. Neutrophil engraftment was defined as the first of 3 consecutive days on which the absolute neutrophil count (ANC) was > 0.5 × 10^9^ without G-CSF support, and platelet (PLT) engraftment was defined as the first day of 7 consecutive days in which PLT was > 20 × 10^9^ without platelet transfusion [13]. Overall survival (OS) was defined as the time in months from the transplant date to the occurrence of death or last follow-up. Progression-free survival (PFS) was defined as the time in months from the transplant date to the occurrence of death, relapse or progression during the follow-up period. Treatment-related mortality (TRM) was defined as death from any cause other than disease recurrence or progression within 100 days after transplantation.

### Statistical analysis

The primary endpoint was OS, and the secondary endpoints were PFS, the CR rate, the progression/relapse rate, and toxicities. The propensity score matching (PSM) method was used to reduce baseline differences between patients in the two groups. The chi-square test or Fisher’s exact test was used to compare the categorical characteristics between the two groups, and continuous variables were analyzed using the independent-samples T test. The Kaplan‒Meier method was used to estimate the OS, PFS, the cumulative progression/relapse rate was used the competing risks analysis, and the log-rank test was used to test the equality of survival curves. Cox proportional hazards regression was used to perform the univariate and multivariate analyses. All analyses were performed using SPSS 26.0 software. The *P* values reported were all two-sided, and the difference was considered statistically significant when the *P* value was lower than 0.05.

## Results

### Patient characteristics

Patient baselines before and after propensity score matching (PSM) are summarized in Supplementary Table S1 and Table 1, respectively. There were 82 patients in the AD-BEAC group and 59 patients in the SD-BEAC group before PSM, and 52 patients in each group were successfully matched. In both groups, most patients were stage III–IV and had an IPI score of 3 or more. There was no difference in clinical characteristics between the two groups (*P *˃ 0.05).

**Table 1:** patient baseline after propensity score

Conditioning regimen	SD-BEAC (%)	AD-BEAC (%)	*P* value
Age, median (range), years	42 (18–69)	40 (18–59)	0.391
≤ 41	30 (57.7)	30 (57.7)	1.000
> 41	22 (42.3)	22 (42.3)	
Gender			
Male	31 (59.6)	33 (63.5)	0.687
Female	21 (40.4)	19 (36.5)	
Disease type			
B cell lymphoma	32 (61.5)	27 (51.9)	0.552
DLBCL	24 (46.2)	17 (32.7)	
Burkitt lymphoma	2 (3.8)	6 (11.5)	
MCL	3 (5.8)	2 (3.8)	
Transformed DLBCL	2 (3.8)	1 (1.9)	
IVLBCL	1 (1.9)	1 (1.9)	
T- and NK-cell lymphoma	20 (38.5)	25 (48.1)	
PTCLs	11(21.2)	14 (26.9)	
NK-T cell lymphoma	5 (9.6)	9 (17.3)	
Lymphoblastic T-cell lymphoma	4 (7.7)	2 (3.8)	
Disease stage			
I–II	12 (23.1)	11 (21.2)	0.813
III–IV	40 (76.9)	41 (78.8)	
IPI scores			
1–3	21 (40.4)	19 (36.5)	0.687
4–5	31 (59.6)	33 (63.5)	
Time from diagnosis to transplant, median (range), months	5 (3–41)	5 (3–15)	0.072
≤ 5	29 (55.8)	28 (53.8)	0.844
> 5	23 (44.2)	24 (46.2)	
Chemotherapy cycles before ASCT, median (range)	4 (3–20)	4 (3–8)	0.157
≤ 4	37 (71.2)	35 (67.3)	0.671
> 4	15 (28.8)	17 (32.7)	
Accumulated dose of anthracycline drugs, mg/m^2^			
≤ 200	35 (67.3)	36 (69.2)	0.833
> 200	17 (32.7)	16 (30.8)	
Disease status before ASCT			
CR	27 (51.9)	28 (53.8)	0.844
PR	25 (48.1)	24 (46.2)	
Newly diagnosed or relapsed/refractory disease			
newly diagnosed	40 (76.9)	41 (78.8)	0.813
relapsed/refractory disease	12 (23.1)	11 (21.2)	

*SD-BEAC* standard-dose BEAC, *AD-BEAC* adjusted-dose BEAC, *DLBCL* diffuse large B-cell lymphoma, *MCL* mantle cell lymphoma, *IVLBCL* intravascular large B-cell lymphoma, *PTCLs* peripheral T-cell lymphomas

### Stem cell mobilization and hematologic recovery

The median count of mononuclear cells (MNCs) was 8.97 × 10^8^/kg (range, 2.17–25.28) in the AD-BEAC group and 9.06 × 10^8^/kg (range, 2.2–19.43) in the SD-BEAC group (*P* = 0.569). The median infused CD34 + cell count was 5.61 × 10^6^/kg (range, 1.78–18.8) in the AD-BEAC group and 4.7 × 10^6^/kg (range, 1–19.53) in the SD-BEAC group (*P* = 0.068). No significant difference was found in either MNC or CD34 + cell counts between the two groups.

Almost all patients had successful ANC and PLT reconstitution. The median time to reconstitution of ANC in the two groups was 11 days (range, 8–20 and 8–18, *P* = 0.845). The median time to reconstitution of PLTs in the two groups was 14 days and 11 days (range, 8–29 and 8–22, *P* = 0.267).

### Response

The transplantation efficacy was assessed 3 months after transplantation, and 47 patients in the AD-BEAC group achieved CR after transplantation, of which 19 patients with PR before transplantation achieved CR after transplantation. In the SD-BEAC group, 39 patients achieved CR after transplantation, and 13 patients with PR before transplantation achieved CR after transplantation. The CR rate of the AD-BEAC conditioning regimen was higher than that of the SD-BEAC conditioning regimen (90.4% vs. 75.0%, *P* = 0.038).

### Progression/relapse rate

**Fig. 1:**
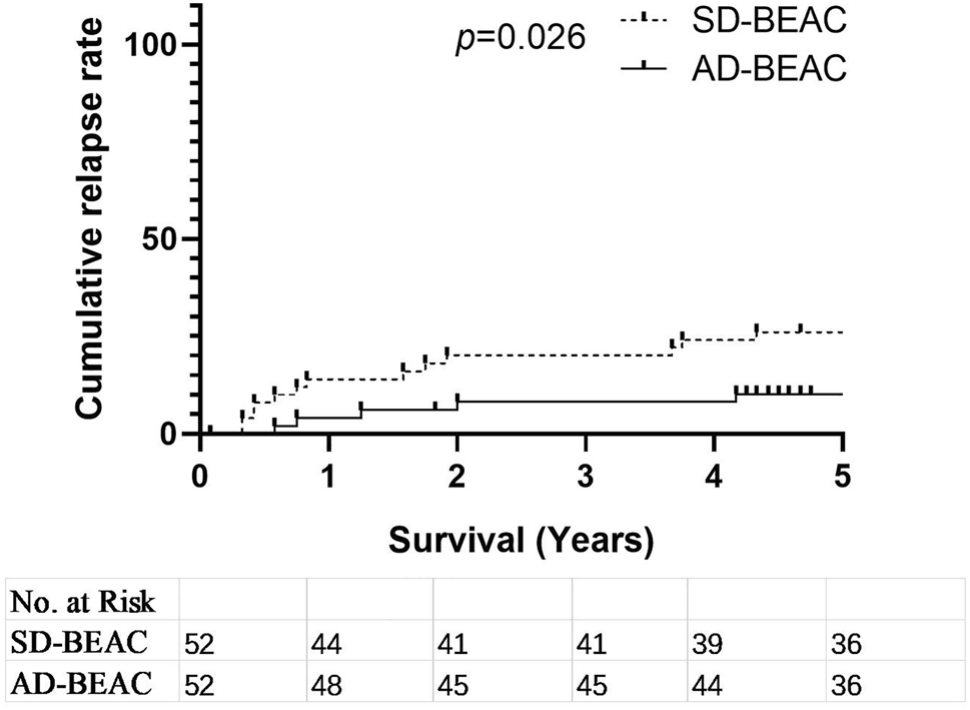
Cumulative relapse rate of SD-BEAC and AD-BEAC conditioning regimen

Since all patients achieved CR/PR before transplantation, we analyzed relapse/progression rates at 3 and 5 years after transplantation. The relapse/progression rate in the AD-BEAC group was much lower than that in the SD-BEAC group (3-year 8% vs. 20%, 5-year 10% vs. 26%, *P* = 0.026, HR 0.3324, 95% CI 1.223–7.399) (Fig. 1). Relapse/progression typically occurred within 2 years after transplantation and mainly occurred in patients who did not achieve CR before transplantation.

### Survival analysis

**Fig. 2:**
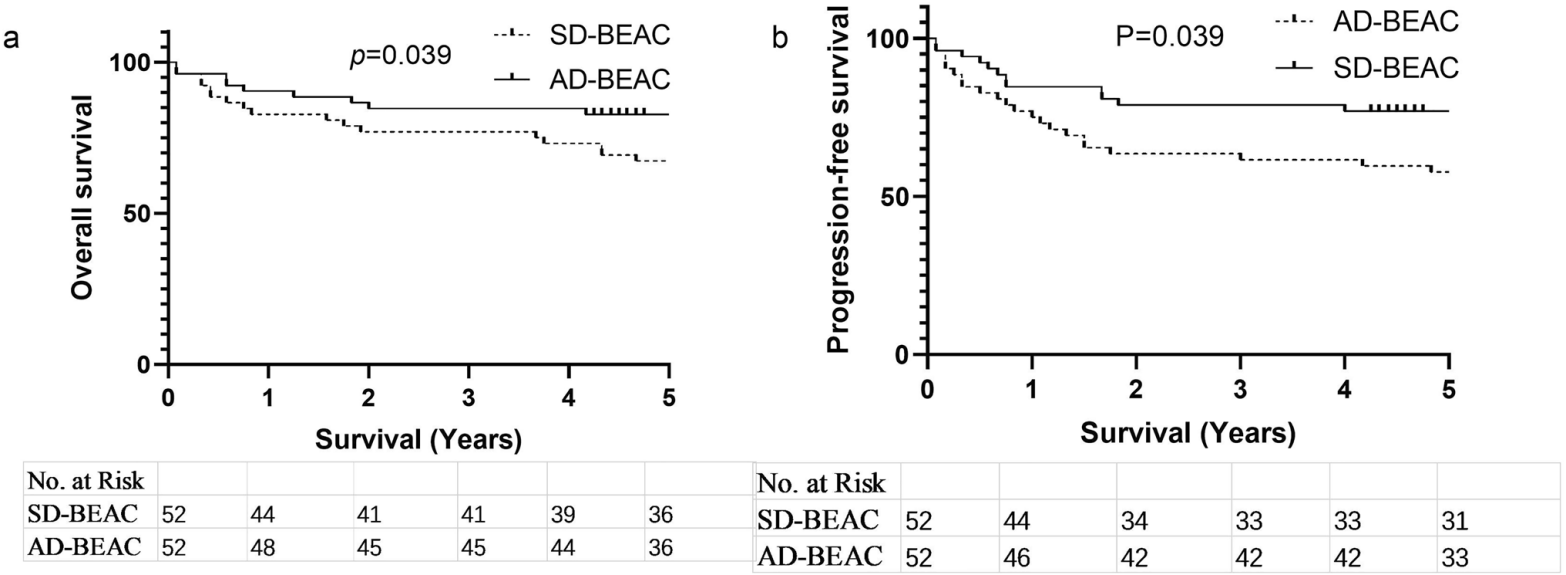
**a** Overall survival (OS) of SD-BEAC and AD-BEAC conditioning regimen, **b** progression-free survival (PFS) of SD-BEAC and AD-BEAC conditioning regimen

The median follow-up time was 85 months in 104 patients after PSM, and the AD-BEAC group had a higher OS and PFS than the SD-BEAC group (5-year OS 82.7% vs. 67.3%, *P* = 0.039, HR 0.4464, 95% CI 0.2128–0.9367; 5-year PFS 76.9% vs. 57.7%, *P* = 0.039, HR 0.4881, 95% CI 0.2489–0.9570) (Fig. 2a, b). In the SD-BEAC group, 15 of 21 patients with recurrence and progression received salvage chemotherapy or targeted therapy, and 4 of them were still alive by the end of follow-up. In the AD-BEAC group, 6 of 8 patients with recurrence and progression received salvage chemotherapy or targeted therapy, and 3 of them were still alive by the end of follow-up.

**Fig. 3:**
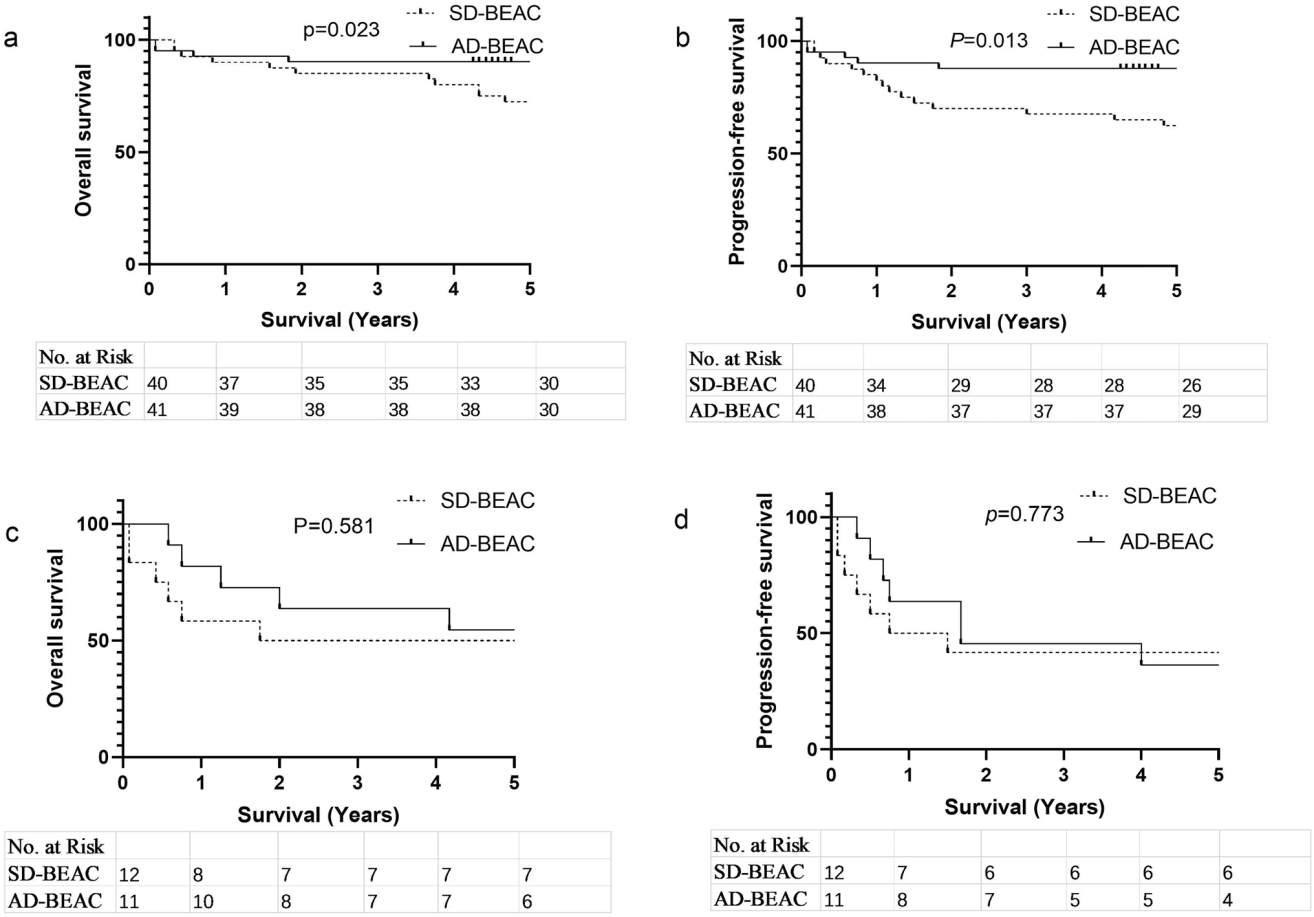
**a** Overall survival (OS) of newly diagnosed high-risk NHL, **b** progression-free survival (PFS) of newly diagnosed high-risk NHL, **c** OS of relapse/refractory NHL, **d** PFS of relapse/refractory NHL

**Fig. 4:**
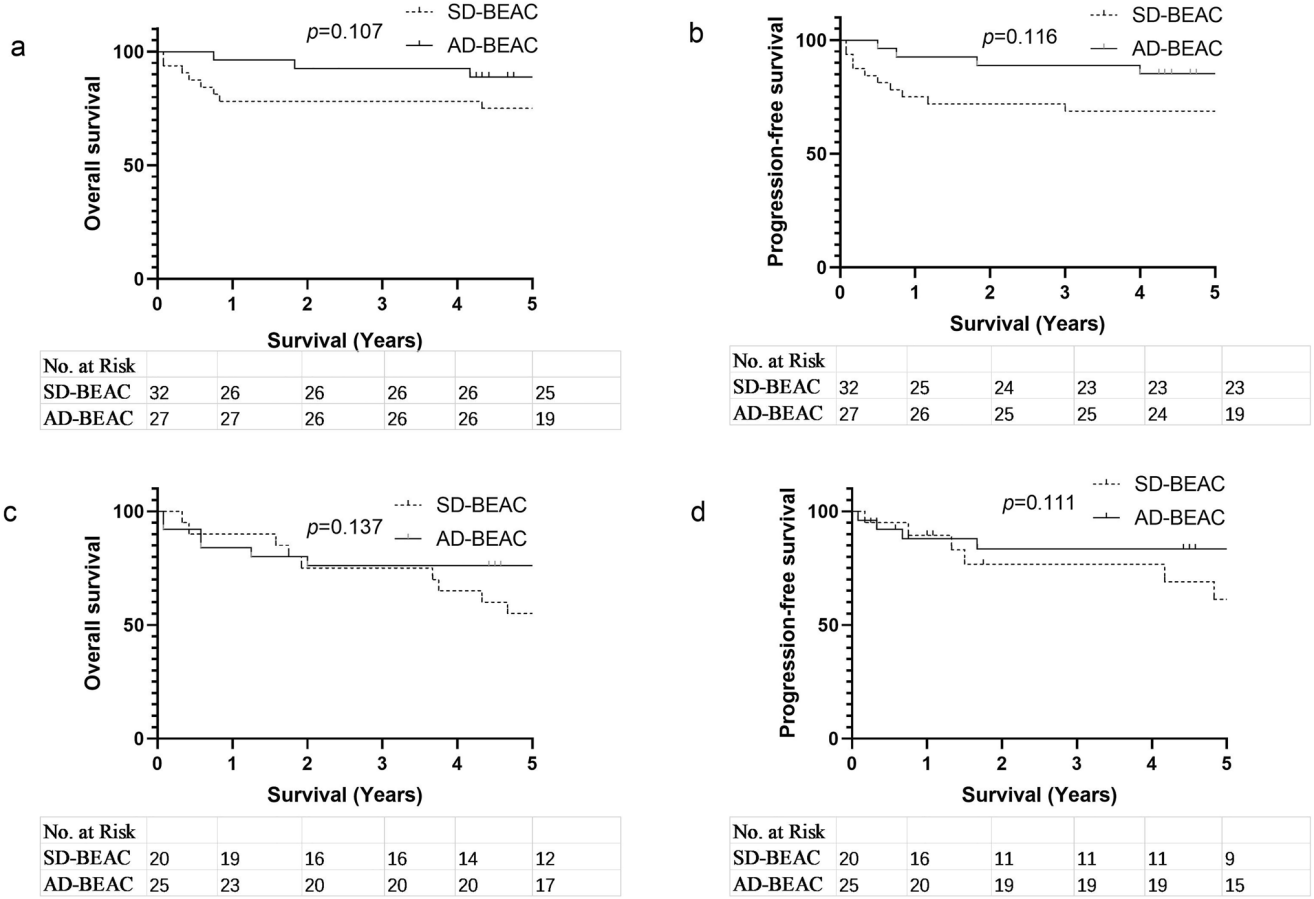
**a** Overall survival (OS) of B-cell lymphoma, **b** progression-free survival (PFS) of B-cell lymphoma, **c** OS of T- and NK-cell lymphoma, **d** PFS of T- and NK-cell lymphoma

We conducted subgroup analysis on different types of lymphoma. For patients with untreated lymphoma, we found that the AD-BEAC group had a higher OS and PFS than the SD-BEAC group (5-year OS 90.2% vs. 72.5%, *P* = 0.023, HR 0.2948, 95% CI 0.1139–0.7631; 5-year PFS 87.8% vs. 62.5%, *P* = 0.013, HR 0.2985 95% CI 0.1241–0.7180) (Fig. 3a, b). However, for relapsed/refractory NHL, there was no significant difference in OS and PFS between the AD-BEAC and SD-BEAC groups (5-year OS 54.5% vs. 50.0%, *P* = 0.581, HR 0.7211, 95% CI 0.2205–2.358; 5-year PFS 36.4% vs. 41.7%, *P* = 0.773, HR 0.8603, 95% CI 0.3009–2.460) (Fig. 3c, d). For patients with B-cell lymphoma, there was no significant difference between the AD-BEAC group and the SD-BEAC group in OS and PFS (5-year OS 88.9% vs. 75.0%, *P* = 0.107, HR 0.3588, 95% CI 0.1156–1.113; 5-year PFS 85.2% vs. 68.8%, *P* = 0.116, HR 0.4085, 95% CI 0.1433–1.165) (Fig. 4a, b). For the patients with T- and NK-cell lymphoma, we found a similar outcome (5-year OS 76.0% vs. 55.0%, *P* = 0.137, HR 0.4738, 95% CI 0.1766–1.271; 5-year PFS 68.0% vs. 40.0%, *P* = 0.111, HR 0.4937, 95% CI 0.2035–1.198) (Fig. 4c, d).

### Univariate and multivariate analyses

Univariate and multivariate analyses were conducted on conditioning regimen, age, disease type, disease stage, IPI scores, time from diagnosis to ASCT, chemotherapy cycles before ASCT, disease status before ASCT and newly diagnosed or relapsed/refractory disease. The univariate analysis showed that the disease type influenced PFS but not OS; additionally, conditioning regimen, disease status before ASCT, and newly diagnosed or relapsed/refractory disease influenced both OS and PFS (*P* < 0.05). However, the multivariate analysis showed that only the conditioning regimen influenced both OS and PFS (Tables 2 and 3).

**Table 2:** Univariate and multivariate analysis of the overall survival

Prognostic variables	Univariate analysis HR (95% CI)	*P*	multivariate analysis HR (95% CI)	*P*
Conditioning regimen (AD-BEAC vs. SD-BEAC)	0.446 (0.202–0.986)	0.046	0.432 (0.194–0.963)	0.040
Age (≤ 41 vs. > 41)	1.452 (0.692–3.047)	0.324	1.765 (0.751–4.149)	0.193
Disease type (B cell lymphoma vs. T- and NK-cell lymphoma)	1.871 (0.202–0.986)	0.101	1.894 (0.801–4.475)	0.146
Disease stage (I–II vs. III–IV)	1.131 (0.459–2.791)	0.789	0.810 (0.300–2.186)	0.677
IPI scores (1–3 vs. 4–5)	1.157 (0.534–2.506)	0.712	1.120 (0.458–2.738)	0.803
Time from diagnosis to ASCT (≤ 5 vs. > 5)	1.733 (0.819–3.664)	0.150	1.230 (0.515–2.938)	0.642
Chemotherapy cycles before ASCT (≤ 4 vs. > 4)	1.932 (0.913–4.085)	0.085	1.596 (0.616–4.134)	0.336
Disease status before ASCT (CR vs. PR)	3.132 (1.465–6.695)	0.003	2.033 (0.842–4.907)	0.115
Newly diagnosed or relapsed/refractory disease (newly diagnosed vs. relapsed/refractory disease)	2.846 (1.330–6.091)	0.007	1.770 (0.744–4.209)	0.197

*HR* hazard ratio, *CI* confidence interval, *IPI* international prognostic index, *ASCT* autologous stem cell transplantation

**Table 3:** Univariate and multivariate analysis of the progression-free survival

Prognostic variables	Univariate analysis HR (95% CI)	*P*	Multivariate analysis HR (95% CI)	*P*
Conditioning regimen (AD-BEAC vs. SD-BEAC)	0.488 (0.241–0.986)	0.045	0.476 (0.234–0.971)	0.041
Age (≤ 41 vs. > 41)	1.338 (0.682–2.623)	0.398	1.582 (0.732–3.418)	0.244
Disease type (B cell lymphoma vs. T- and NK-cell lymphoma)	2.062 (1.041–4.086)	0.038	2.149 (0.978–4.721)	0.057
Disease stage (I–II vs. III–IV)	1.532 (0.634–3.702)	0.343	1.183 (0.457–3.066)	0.729
IPI scores (1–3 vs. 4–5)	1.401 (0.683–2.875)	0.357	1.518 (0.683–3.373)	0.306
Time from diagnosis to ASCT (≤ 5 vs. > 5)	1.670 (0.848–3.288)	0.138	1.245 (0.558–2.776)	0.593
Chemotherapy cycles before ASCT (≤ 4 vs. > 4)	1.826 (0.922–3.618)	0.084	1.329 (0.556–3.180)	0.522
Disease status before ASCT (CR vs. PR)	3.006 (1.515–5.961)	0.002	1.948 (0.888–4.274)	0.096
Newly diagnosed or relapsed/refractory disease (newly diagnosed vs. relapsed/refractory disease)	3.302 (1.662–6.560)	0.001	2.050 (0.921–4.561)	0.079

*HR* hazard ratio, *CI* confidence interval, *IPI* international prognostic index, *ASCT* autologous stem cell transplantation

### Toxicity

Major transplantation-related complications are summarized in Table 4. Fever, vomiting, diarrhea, and hepatobiliary disorders were the most common nonhematological toxicities. The AD-BEAC group had a lower incidence of mucositis oral (5.8% vs. 23.1%, *P* = 0.023) and cardiac disorders (1.9% vs. 15.3%, *P* = 0.048). mucositis oral in the AD-BEAC group were all grade I–II, and 5 of 12 patients with mucositis oral in the SD-BEAC group were grade III. A total of 8 patients in the SD-BEAC group experienced cardiotoxicities, including 2 cases of grade I atrial fibrillation, 2 cases of grade I palpitations, 1 case of grade I heart failure, 1 case of grade I pericardial effusion, and 2 cases of grade III heart failure. However, only 1 case of grade I atrial fibrillation occurred in the AD-BEAC group. There was no difference for other adverse events. The TRM in both the AD-BEAC and SD-BEAC groups was 3.8% (2/52). The rate of secondary malignancies between the two groups was 1.9% (1/52); one patient in the AD-BEAC group developed lung cancer 4 years after transplantation, and one patient in the SD-BEAC group developed parotid gland cancer 5 years after transplantation.

**Table 4:** Transplant-related toxicity

Toxicities	Grade	SD-BEAC (%)	AD-BEAC (%)	*P* value
Fever				
	I–II	39 (75.0)	46 (88.5)	0.183
	III–IV	6 (11.5)	2 (3.8)	
Bacteremia				
	I–II	10 (19.2)	8 (15.4)	0.604
	III–IV	0 (0.0)	0 (0.0)	
Nausea				
	I–II	40 (76.9)	36 (69.2)	0.672
	III–IV	4 (7.7)	5 (9.6)	
Vomiting				
	I–II	23 (44.2)	21 (40.4)	0.700
	III–IV	6 (11.5)	9 (17.3)	
Mucositis oral				
	I–II	7 (13.5)	3 (5.8)	0.023
	III–IV	5 (9.6)	0 (0.0)	
Hepatobiliary disorders				
	I–II	18 (34.6)	18 (34.6)	1.000
	III–IV	1 (1.9)	1 (1.9)	
Renal and urinary disorders				
	I–II	3 (5.8)	1 (1.9)	0.610
	III–IV	0 (0.0)	0 (0.0)	
Cardiac disorders				
	I–II	6 (11.5)	1 (1.9)	0.048
	III–IV	2 (3.8)	0 (0.0)	

*SD–BEAC* standard-dose BEAC, *AD-BEAC* adjusted-dose BEAC

## Discussion

Despite the advent of novel agents, ASCT remains the standard care for patients with relapsed/refractory lymphoma. For high-risk NHL, studies have also shown benefits from upfront consolidative ASCT [14–17]. The conditioning regimen is an important part of ASCT, and an ideal conditioning regimen should eliminate tumor cells while also demonstrating controllable toxicity. However, the best conditioning regimen for lymphoma ASCT has not been well defined. The BEAM and BEAC regimens seem to be the most commonly used, and because of the supply problem of melphalan, the BEAC regimen is more commonly used in China. In the standard-dose BEAC conditioning regimen, the dose of cyclophosphamide is high. In NHL patients who had previously received high cumulative doses of cyclophosphamide, a conditioning regimen containing high-dose cyclophosphamide increases the risk of cardiotoxicity. The second-line regimen containing medium- or high-dose cytarabine has proven to be effective and non-cross-resistant for relapsed/refractory lymphoma. Therefore, we explored an optimized BEAC (AD-BEAC) conditioning regimen with a reduced dose of cyclophosphamide and an increased dose of cytarabine in NHL.

After transplantation, more patients in the AD-BEAC group with PR achieved CR than in the SD-BEAC group, and the CR rate of the AD-BEAC group was higher than that of the SD-BEAC group. Additionally, the relapse/progression rate of the AD-BEAC group was lower than that of the SD-CEAC group. In an earlier study, O et al. reported that the relapse/progression rate was 49% for NHL after ASCT, and the median survival was 7.5 months after relapse or progression [18]. Another phase II study reported a relapse/progression rate of 19.4% with a new conditioning regimen of BEB (bendamustine, etoposide, and busulfan) for ASCT in NHL [19]. The relapse/progression rate of our AD-BEAC conditioning regimen was lower than that of the above study. In the multivariate Cox regression analysis by David et al. [20], disease status was the most powerful predictor for OS, PFS and relapse. Similarly, we consider that the AD-BEAC conditioning regimen can enable more patients to achieve CR, which may further reduce the relapse/progression rate.

Jo et al. [21] reported that the TRM of the BEAM or BEAC conditioning regimen was 7.1%, and in a multicenter phase II study with the BuCyE conditioning regimen for ASCT in HL and NHL, the reported TRM was 4.5% [22]. In our study, the TRM of the AD-BEAC conditioning regimen was 3.8%, which was lower than that reported previously. Cardiotoxicity is a severe complication that may be associated with high-dose cyclophosphamide, and this decreased significantly when we reduced the dose of cyclophosphamide in the adjusted-dose BEAC regimen. However, it should be noted that the influence of prior anthracycline exposure could not be evaluated. We also found that the incidence of oral mucositis in the AD-BEAC conditioning group was lower than that in the SD-BEAC conditioning group and that there was no severe mucositis oral. Additionally, the incidence of grade III-IV fever and bacteremia was slightly lower in the AD-BEAC group than in the SD-BEAC group. Infections associated with mucositis lesions can cause life-threatening systemic sepsis during periods of profound immunosuppression [23], and severe mucositis is associated with reduced survival after ASCT for lymphoid malignancies [24]. The mucositis also results in impaired nutrient and fluid intake, and patients with moderate or severe malnourishment have a higher incidence of bacteremia at 30 days post-ASCT [25]. In two observational prospective multicenter studies, a total of 720 patients underwent ASCT, and 20% of patients developed bacteremia. A duration of neutropenia exceeding 9 days is the only risk factor for bacteremia. However, the increase in the incidence rate of bacteremia had no effect on the overall mortality and infection-related mortality [26]. In our study, the incidence of bacteremia in the AD-BEAC group was 15.4%, and in the SD-BEAC group, it was 19.2%, which was similar to the results of the previous study.

When we adjusted the dose of the BEAC regimen, the 5-year OS and PFS were significantly improved, and our results were superior to those of previously reported BEAM (5-year OS 77.8%, 5-year PFS 66.7%) and BEAC (5-year OS 81.8%, 5-year PFS 67.5%) conditioning regimens [3, 4]. Additionally, both univariate and multivariate analyses showed that the conditioning regimen was a prognostic factor affecting both OS and PFS. Dose optimization can be used to explore the ideal conditioning regimen. Factors such as age, disease status at transplant, histology, and IPI scores have been influential covariates across the literature. In this study, the multivariate analysis showed no impact of these various factors, probably due to the limited sample size.

The subgroup analysis showed that patients with newly diagnosed high-risk NHL who received a conditioning regimen of AD-BEAC had better OS and PFS than those who received the SD-BEAC conditioning regimen. It should also be noted that the majority of patients are not eligible for ASCT due to refractory disease or age/comorbidities [27]. Therefore, upfront ASCT consolidation therapy may improve the prognosis of high-risk NHL patients, and the AD-BEAC conditioning regimen is more effective for high-risk NHL.

For patients with relapsed/refractory NHL, there was no significant difference in OS or PFS between our two conditioning regimens. This result suggests that adjusting the dosage of the BEAC conditioning regimen alone could not improve the outcome of relapsed/refractory NHL. Except for the limited number of patients with relapsed and refractory lymphoma enrolled in our study, the critical reason may be the presence of gene mutations with poor prognosis in relapsed/refractory NHL, which typically exhibits resistance to almost all traditional chemotherapy drugs. Thus, there is an urgent need to explore new methods for patients with relapsed/refractory NHL, such as combining treatment with novel drugs with different mechanisms. A phase II clinical study evaluated the combination of chidamide-cladribine-gemcitabine-busulfan (ChiCGB) as a novel conditioning regimen in patients with high-risk or relapse/refractory lymphoma. At a median follow-up of 35.4 months, the estimated 4-year PFS and OS were 80.6% and 86.1%, respectively. The PFS and OS of high-risk patients in CR1 and relapsed/refractory patients in CR2/3 were similar [28]. Additionally, in this study, 86.7% of the patients achieved CR at the time of transplantation. In recent years, with the development of chimeric antigen receptor (CAR) T-cell therapy, it has become a potential treatment for NHL. Anti-CD19 CAR-T cells created a sustainable recovery in 40% of chemotherapy-resistant DLBCL, HGBCL, and PMBCL patients who had not previously received any treatment options. Additionally, these products are currently used in patients with aggressive lymphoma who have relapsed after at least 2 previous treatment lines. In addition, clinical trials of anti-CD19 CAR-T cells in patients with DLBCL are being considered as a treatment option in the first recurrence [29]. Therefore, for relapsed/refractory NHL, we hope that novel agents can play a synergistic role in disease salvage to allow more patients to reach CR before transplantation in combination with the conditioning regimen of ASCT to further improve efficacy or new technical means, such as CAR-T treatment.

Our research showed that the optimized BEAC conditioning regimen can further improve the OS and PFS of ASCT for NHL. The optimized BEAC conditioning regimen of upfront ASCT was more beneficial, especially for patients with high-risk NHL. However, this study had limitations, as it was a retrospective study with a limited number of cases. In the future, we will carry out an RCT study to further evaluate its efficacy and explore novel agent combination therapies for relapsed/refractory NHL.

### Supplementary Information

Below is the link to the electronic supplementary material.

Supplementary file1 (DOCX 17 KB)

Supplementary file2 (DOCX 18 KB)

Supplementary file3 (DOCX 18 KB)

## Data Availability

The authors confirm that the data supporting the findings of this study are available within the article and its supplementary materials.
