# Supplementary material for: Optimized BEAC conditioning regimen improves clinical outcomes of autologous hematopoietic stem cell transplantation in non-Hodgkin lymphomas
Source: Int J Hematol. 2024 Apr 8;120(1):96–105. doi: 10.1007/s12185-024-03755-7 (PMC11226560; doi:10.1007/s12185-024-03755-7)
Supplement: Supplementary file 1 — Supplementary file1 (DOCX 17 KB) [file 12185_2024_3755_MOESM1_ESM.docx]

Table S1. Patient Baseline Before Propensity Score

| Conditioning regimen | SD-BEAC (%) | AD-BEAC (%) | P value |
| --- | --- | --- | --- |
| Age, median (range), years  ≤41  ＞41 | 39 (18-69)  35 (59.3)  24 (60.7) | 43 (18-63)  38 (46.3)  44 (53.7) | 0.626  0.128 |
| Gender  Male  Female | 37 (62.7)  22 (37.3) | 47 (57.3)  35 (42.7) | 0.520 |
| Disease type  B cell lymphoma  T- and NK-cell lymphoma | 36 (61.0)  23 (39.0) | 54 (65.9)  28 (34.1) | 0.555 |
| Disease stage  Ⅰ-Ⅱ  Ⅲ-Ⅳ | 18 (30.5)  41 (69.5) | 22 (26.8)  60 (73.2) | 0.633 |
| IPI scores  1-3  4-5 | 25 (42.4)  34 (57.6) | 31 (37.8)  51 (62.2) | 0.584 |
| Time from diagnosis to transplant, median (range), months  ≤5  ＞5 | 5 (3-41)  34 (57.6)  25 (42.4) | 5 (3-19)  42 (51.2)  40 (48.8) | 0.054  0.451 |
| Chemotherapy cycles before ASCT, median (range)  ≤4  ＞4 | 4 (3-20)  42 (71.2)  17 (28.8) | 4 (3-11)  54 (65.9)  28 (34.1) | 0.134  0.503 |
| Disease status before ASCT  CR  PR | 27 (45.8)  32 (54.2) | 44 (53.7)  38 (46.3) | 0.355 |
| Newly diagnosed or relapsed/refractory disease  newly diagnosed  relapsed/refractory disease | 46 (78.0)  13 (22.0) | 67 (81.7)  15 (18.3) | 0.583 |

^SD-BEAC: standard-dose BEAC; AD-BEAC: adjusted-dose BEAC^
