# Supplementary material for: Optimized BEAC conditioning regimen improves clinical outcomes of autologous hematopoietic stem cell transplantation in non-Hodgkin lymphomas
Source: Int J Hematol. 2024 Apr 8;120(1):96–105. doi: 10.1007/s12185-024-03755-7 (PMC11226560; doi:10.1007/s12185-024-03755-7)
Supplement: Supplementary file 2 — Supplementary file2 (DOCX 18 KB) [file 12185_2024_3755_MOESM2_ESM.docx]

Table S2. Newly Diagnosed Patient Baseline After Propensity Score

| Conditioning regimen | SD-BEAC (%) | AD-BEAC (%) | P value |
| --- | --- | --- | --- |
| Age, median (range), years  ≤40  ＞40 | 39 (18-69)  22 (55.0)  18 (45.0) | 41 (18-59)  20 (48.8)  21 (51.2) | 0.739  0.575 |
| Gender  Male  Female | 23 (57.5)  17 (42.5) | 26 (63.4)  15 (36.6) | 0.586 |
| Disease type  B cell lymphoma  DLBCL  Transformed DLBCL  MCL  Burkitt lymphoma  IVLBCL  T- and NK-cell lymphoma  PTCLs  NK-T cell lymphoma  Lymphoblastic T-cell lymphoma | 25 (62.5)  20 (50.0)  2 (5.0)  2 (5.0)  0 (0.0)  1 (2.5)  15 (37.5)  9 (22.5)  3 (7.5)  3 (7.5) | 21 (51.2)  14 (34.1)  2 (4.9)  1 (2.4)  3 (7.3)  1 (2.4)  20 (48.8)  12 (29.2)  7 (17.1)  1 (2.4) | 0.388 |
| Disease stage  Ⅰ-Ⅱ  Ⅲ-Ⅳ | 9 (22.5)  31 (77.5) | 7 (17.1)  34 (82.9) | 0.540 |
| IPI scores  1-3  4-5 | 13 (32.5)  27 (67.5) | 11 (26.8)  30 (73.2) | 0.576 |
| Time from diagnosis to transplant, median (range), months  ≤5  ＞5 | 5 (3-10)  26 (65.0)  14 (35.0) | 5 (4-10)  24 (58.5)  17 (41.5) | 0.623  0.550 |
| Chemotherapy cycles before ASCT, median (range)  ≤4  ＞4  Accumulated dose of anthracycline drugs, mg/m^2^  ≤200  ＞200 | 4 (3-6)  34 (85.0)  6 (15.0)  33 (82.5)  7 (17.5) | 4 (3-6)  30 (73.2)  11 (26.8)    30 (73.2)  11 (26.8) | 0.310  0.191    0.313 |
| Disease status before ASCT  CR  PR | 23 (57.5)  17 (42.5) | 25 (61.0)  16 (39.0) | 0.750 |

^SD-BEAC: standard-dose BEAC; AD-BEAC: adjusted-dose BEAC; DLBCL: diffuse large B-cell lymphoma; MCL: mantle cell lymphoma; IVLBCL: intravascular large B-cell lymphoma; PTCLs: peripheral T-cell lymphomas.^
