# Supplementary material for: Optimized BEAC conditioning regimen improves clinical outcomes of autologous hematopoietic stem cell transplantation in non-Hodgkin lymphomas
Source: Int J Hematol. 2024 Apr 8;120(1):96–105. doi: 10.1007/s12185-024-03755-7 (PMC11226560; doi:10.1007/s12185-024-03755-7)
Supplement: Supplementary file 3 — Supplementary file3 (DOCX 18 KB) [file 12185_2024_3755_MOESM3_ESM.docx]

Table S3. Relapsed/Refractory Patient Baseline After Propensity Score

| Conditioning regimen | SD-BEAC (%) | AD-BEAC (%) | P value |
| --- | --- | --- | --- |
| Age, median (range), years  ≤33  ＞33 | 35 (22-59)  6 (50.0)  6 (50.0) | 31 (18-58)  6 (54.5)  5 (45.5) | 0.330  0.827 |
| Gender  Male  Female | 8 (66.7)  4 (33.3) | 7 (63.6)  4 (36.4) | 1.000 |
| Disease type  B cell lymphoma  DLBCL  Transformed DLBCL  MCL  Burkitt lymphoma  IVLBCL  T- and NK-cell lymphoma  PTCLs  NK-T cell lymphoma  Lymphoblastic T-cell lymphoma | 7 (58.3)  4 (33.3)  0 (0.0)  1 (8.3)  2 (16.7)  0 (0.0)  5 (41.7)  2 (16.7)  2 (16.7)  1 (8.3) | 6 (54.5)  3 (27.3)  0 (0.0)  0 (0.0)  3 (27.3)  0 (0.0)  5 (45.5)  2 (18.2)  2 (18.2)  1 (9.1) | 0.935 |
| Disease stage  Ⅰ-Ⅱ  Ⅲ-Ⅳ | 3 (25.0)  9 (75.0) | 4 (29.8)  7 (63.6) | 0.890 |
| IPI scores  1-3  4-5 | 8 (66.7)  4 (33.3) | 8 (72.7)  3 (27.3) | 1.000 |
| Time from diagnosis to transplant, median (range), months  ≤7  ＞7 | 12 (3-41)  4 (33.3)  8 (66.7) | 6 (3-15)  9 (81.8)  2 (18.2) | 0.298  0.055 |
| Chemotherapy cycles before ASCT, median (range)  ≤7  ＞7  Accumulated dose of anthracycline drugs, mg/m^2^  ≤300  ＞300 | 8 (4-20)  3 (25.0)  9 (75.0)  5 (41.7)  7 (58.3) | 5 (3-8)  5 (45.5)  6 (54.6)  8 (72.7)  3 (27.3) | 0.389  0.555  0.280 |
| Disease status before ASCT  CR  PR | 4 (33.3)  8 (66.7) | 3 (27.3)  8 (72.7) | 1.000 |

^SD-BEAC: standard-dose BEAC; AD-BEAC: adjusted-dose BEAC; DLBCL: diffuse large B-cell lymphoma; MCL: mantle cell lymphoma; IVLBCL: intravascular large B-cell lymphoma; PTCLs: peripheral T-cell lymphomas.^
